# Supplementary material for: Asymmetric subgenomic chromatin architecture impacts on gene expression in resynthesized and natural allopolyploid Brassica napus
Source: Commun Biol. 2022 Jul 29;5:762. doi: 10.1038/s42003-022-03729-7 (PMC9338098; doi:10.1038/s42003-022-03729-7)
Supplement: Supplementary file 2 — Supplementary Information [file 42003_2022_3729_MOESM2_ESM.doc]

**Asymmetric** **subgenomic chromatin architecture impacts on gene expression in resynthesized and natural allopolyploid *Brassica napus***

**
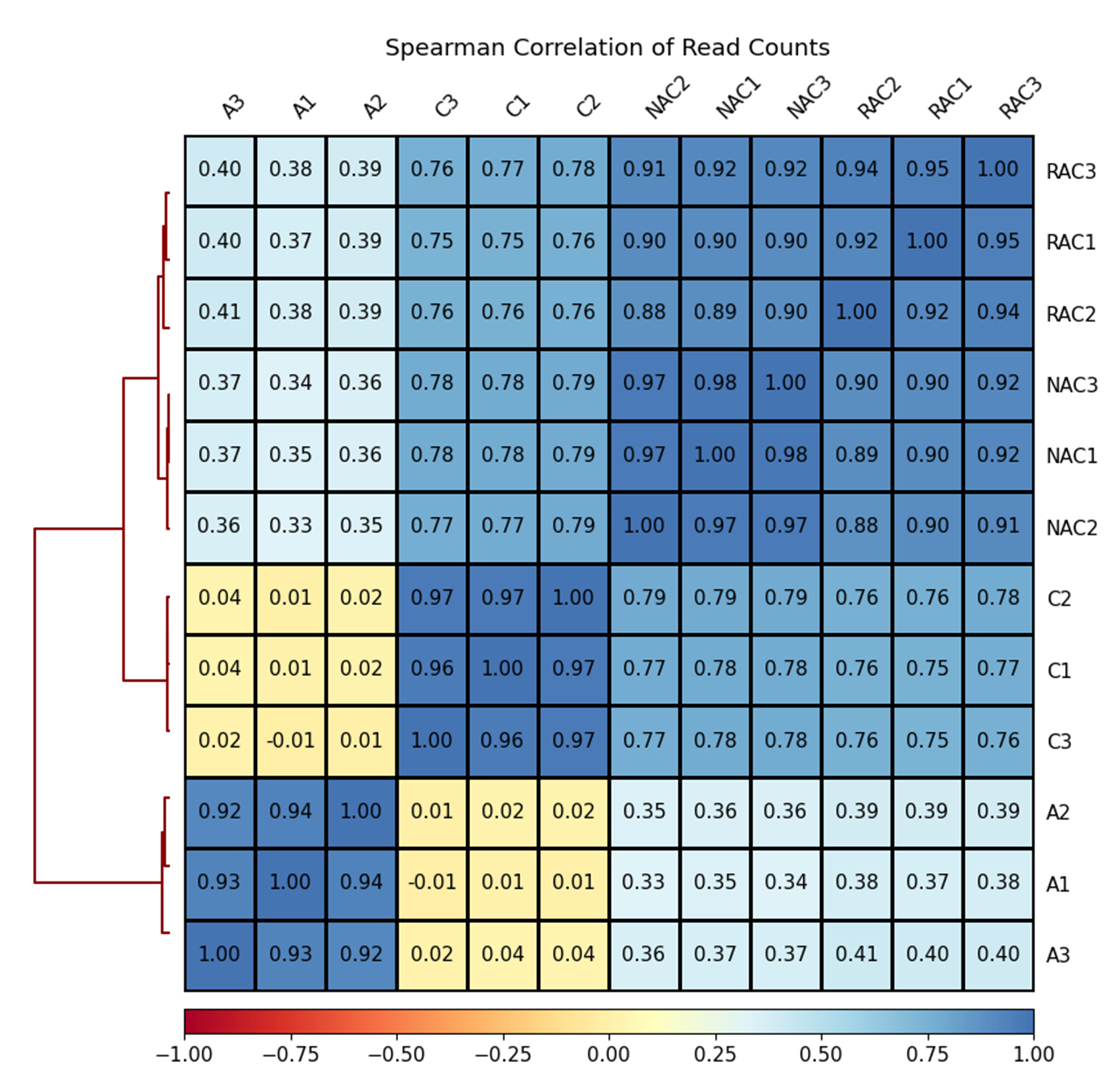
**

**Supplementary Fig. 1 Pearson correlation analysis of three biological replicates of ATAC-Seq in leaves of A, C, RAC, and NAC, respectively.** A, *B. rapa*; C, *B. oleracea*; RAC, resynthesized *B. napus*; NAC, natural *B. napus*.

**
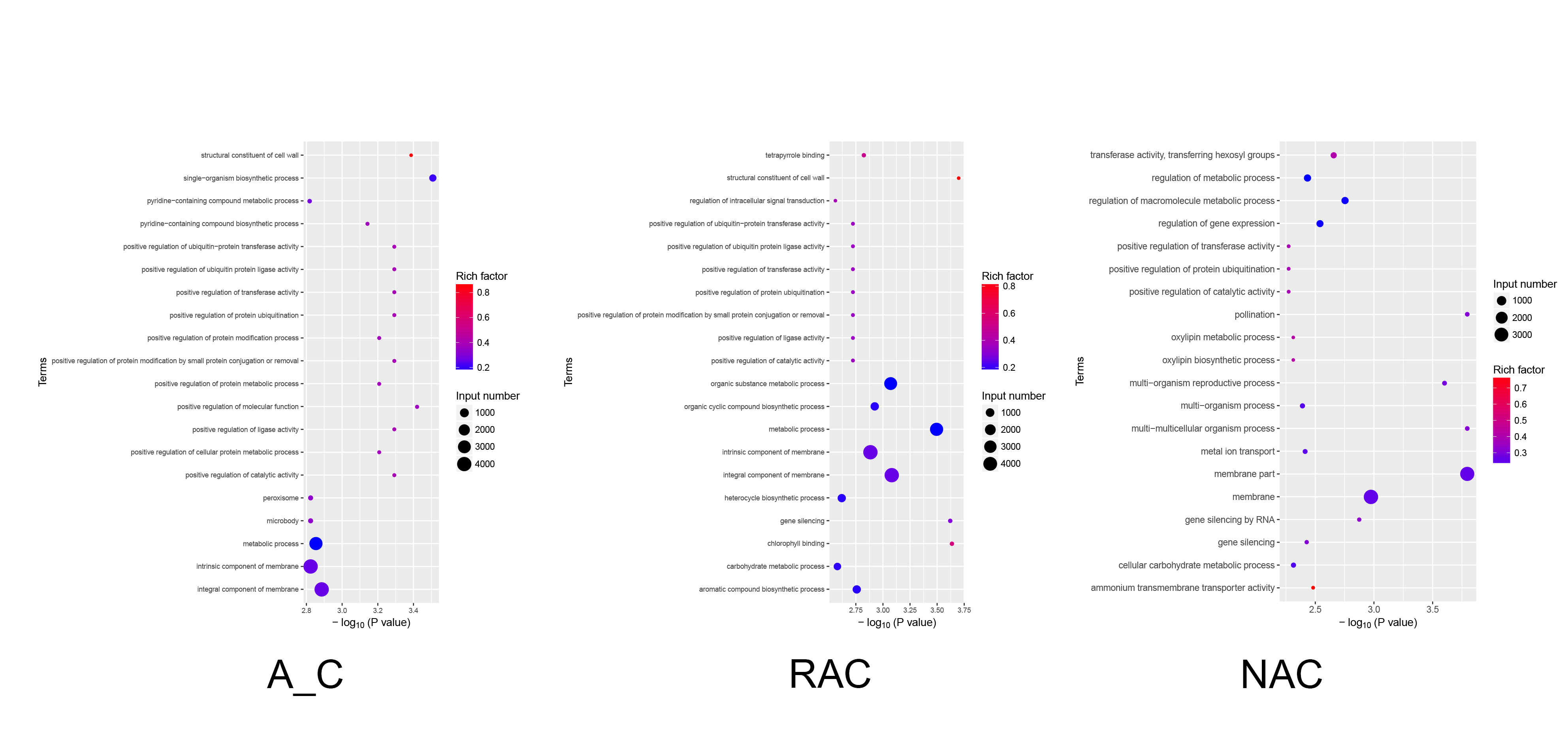
**

**Supplementary Fig. 2** **GO analysis of genes associated with ACRs in three genotypes.** A_C, *in silico* ‘hybrid’; RAC, resynthesized *B. napus*; NAC, natural *B. napus*.

**
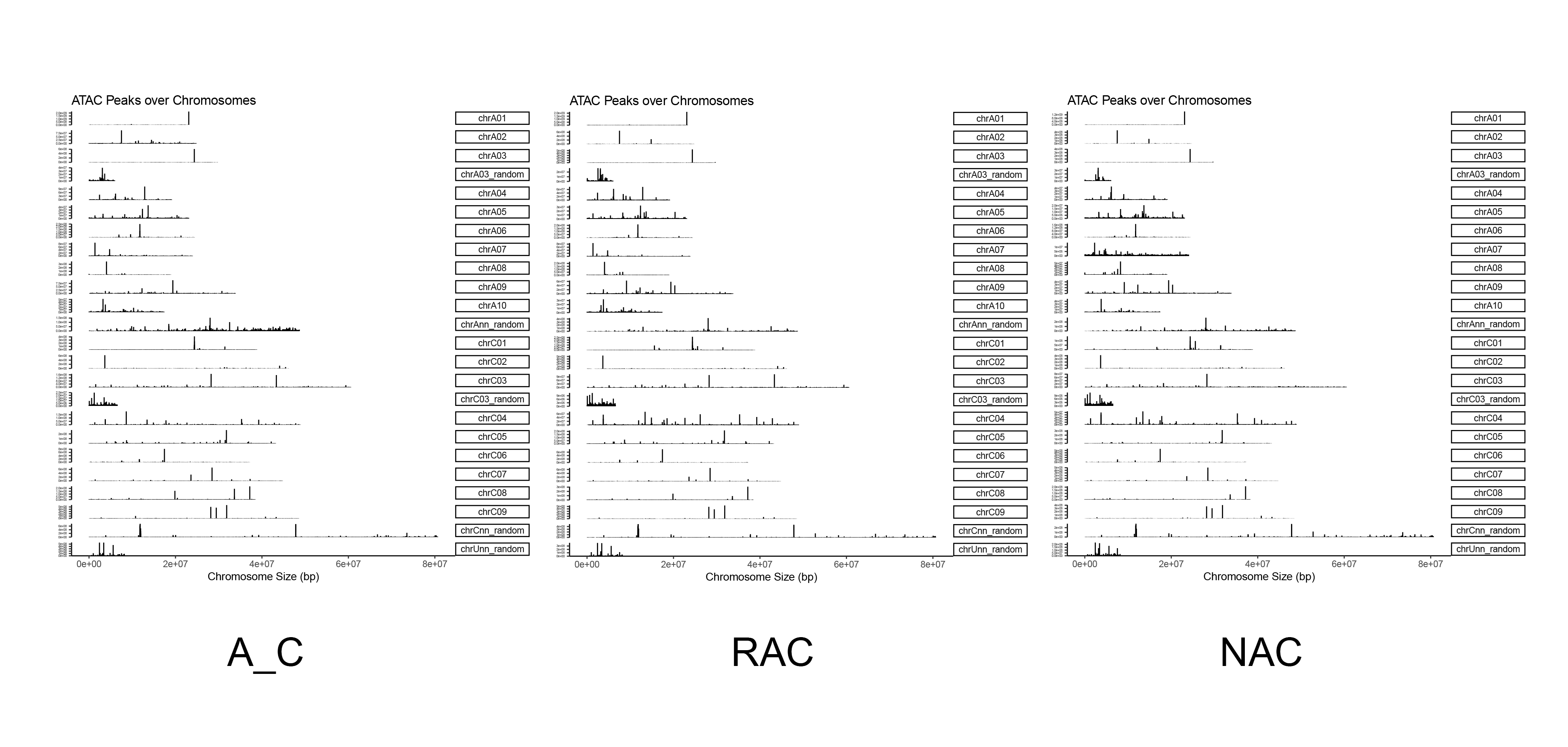
**

**Supplementary Fig. 3 ATAC-Seq signal along chromosomes**. A_C, *in silico* ‘hybrid’. RAC, resynthesized *B. napus*; NAC, natural *B. napus*.

**
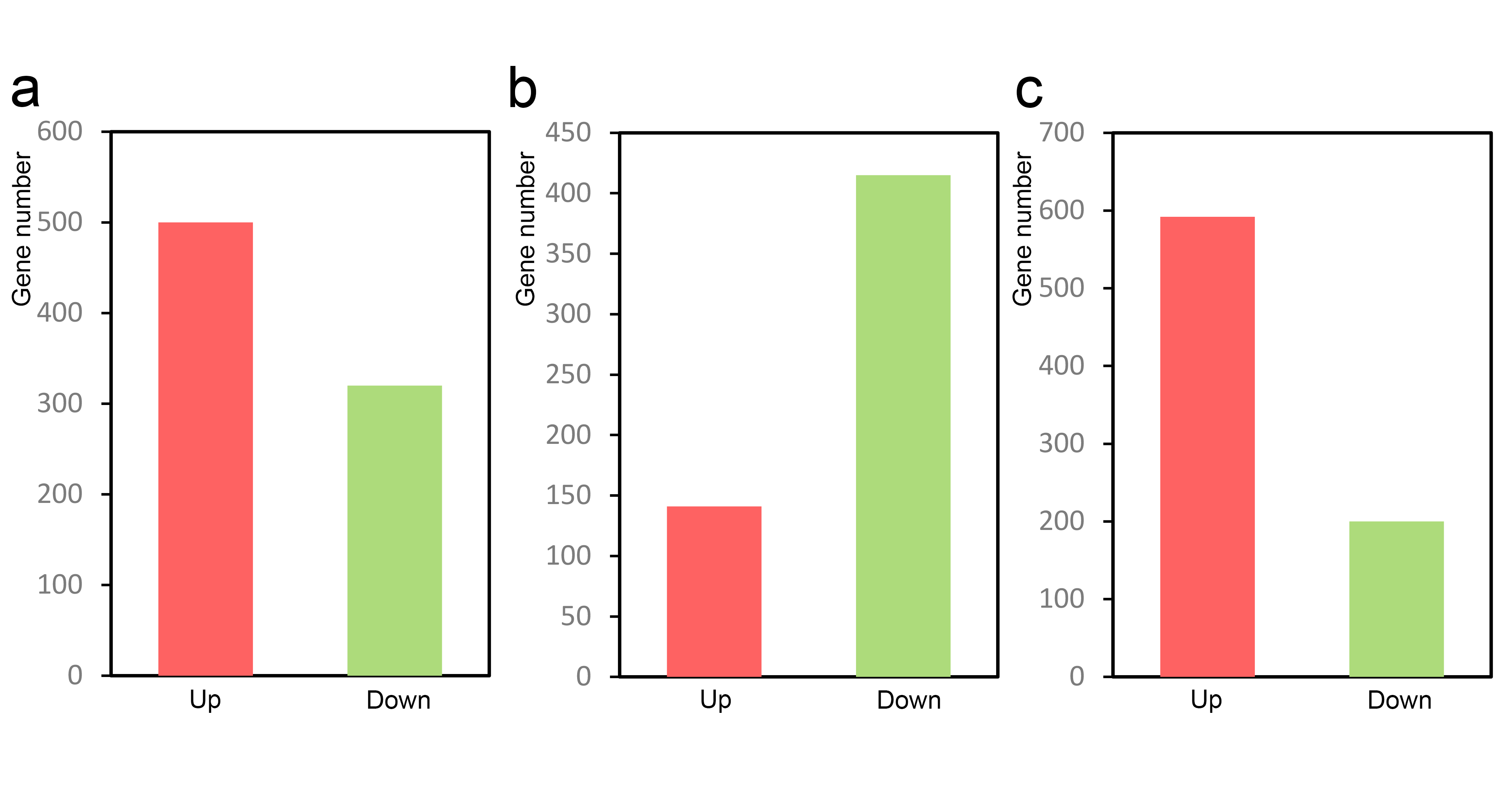
**

**Supplementary Fig. 4 Numbers of genes associated with different enriched ACRs among genotypes.** Numbers of genes associated with different enriched ACRs in (**a**) A_C *vs*. RAC; (**b**) RAC *vs*. NAC; (**c**) A_C *vs*. NAC. A_C, *in silico* ‘hybrid’; RAC, resynthesized *B. napus*; NAC, natural *B. napus*.

**
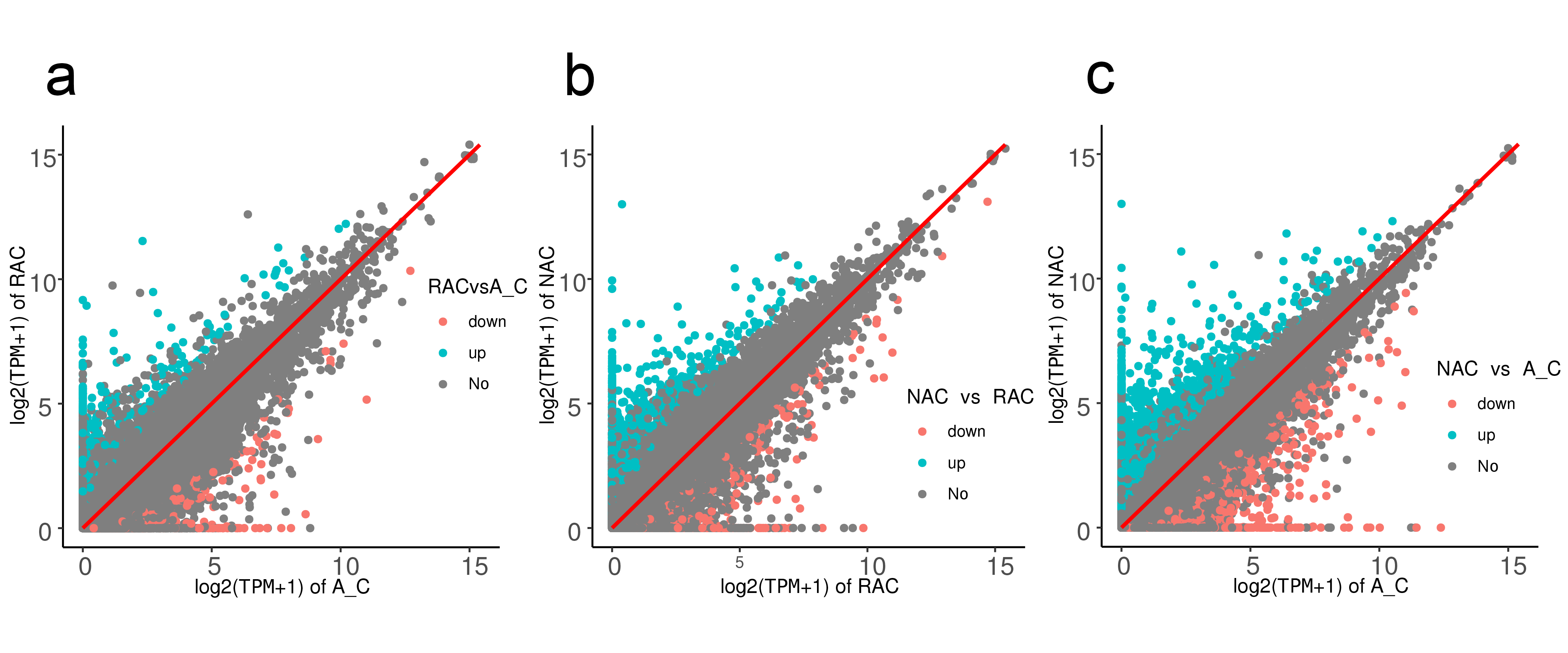
**

**Supplementary Fig. 5 Gene expression change in three genotypes of *B. napus*.** **a** Distribution of gene expression levels in RAC *vs*. A_C. **b** Distribution of gene expression levels in NAC *vs*. RAC. **c** Distribution of gene expression levels in NAC *vs*. A_C. A_C, *in silico* ‘hybrid’; RAC, resynthesized *B. napus*; NAC, natural *B. napus*.

**
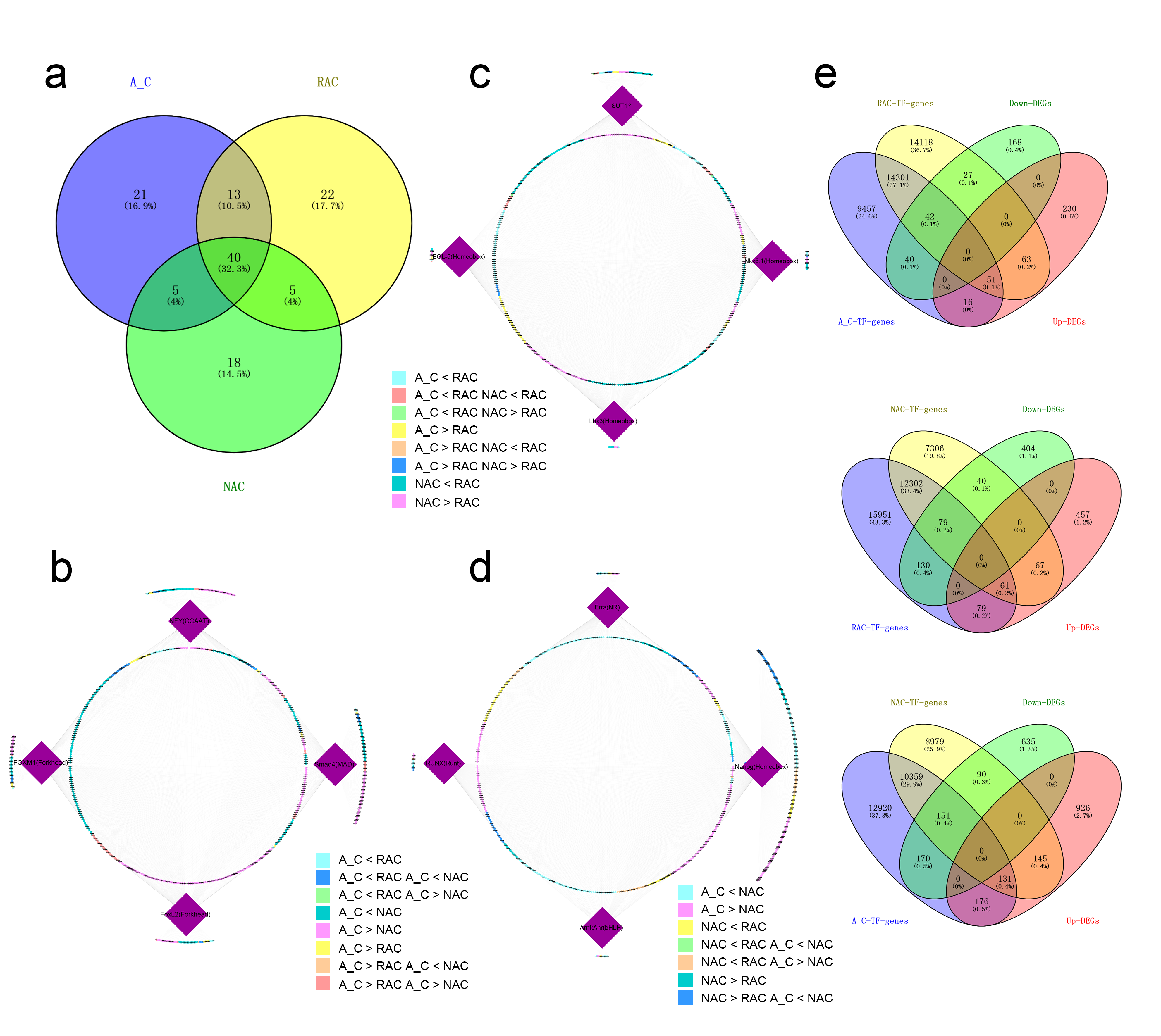
**

**Supplementary Fig. 6 Identification of *cis* and *trans* regulators and TFs regulatory networks.** **a** Overlap of putative TF families. **b** Top 4 A_C-unique TFs regulatory networks. **c** Top 4 RAC-unique TFs regulatory networks. **d** Top 4 NAC-unique TFs regulatory networks. **e** Overlap of genotype-unique TFs targeted genes and DEGs. A_C, *in silico* ‘hybrid’; RAC, resynthesized *B. napus*; NAC, natural *B. napus*; TFs, transcription factors.

**
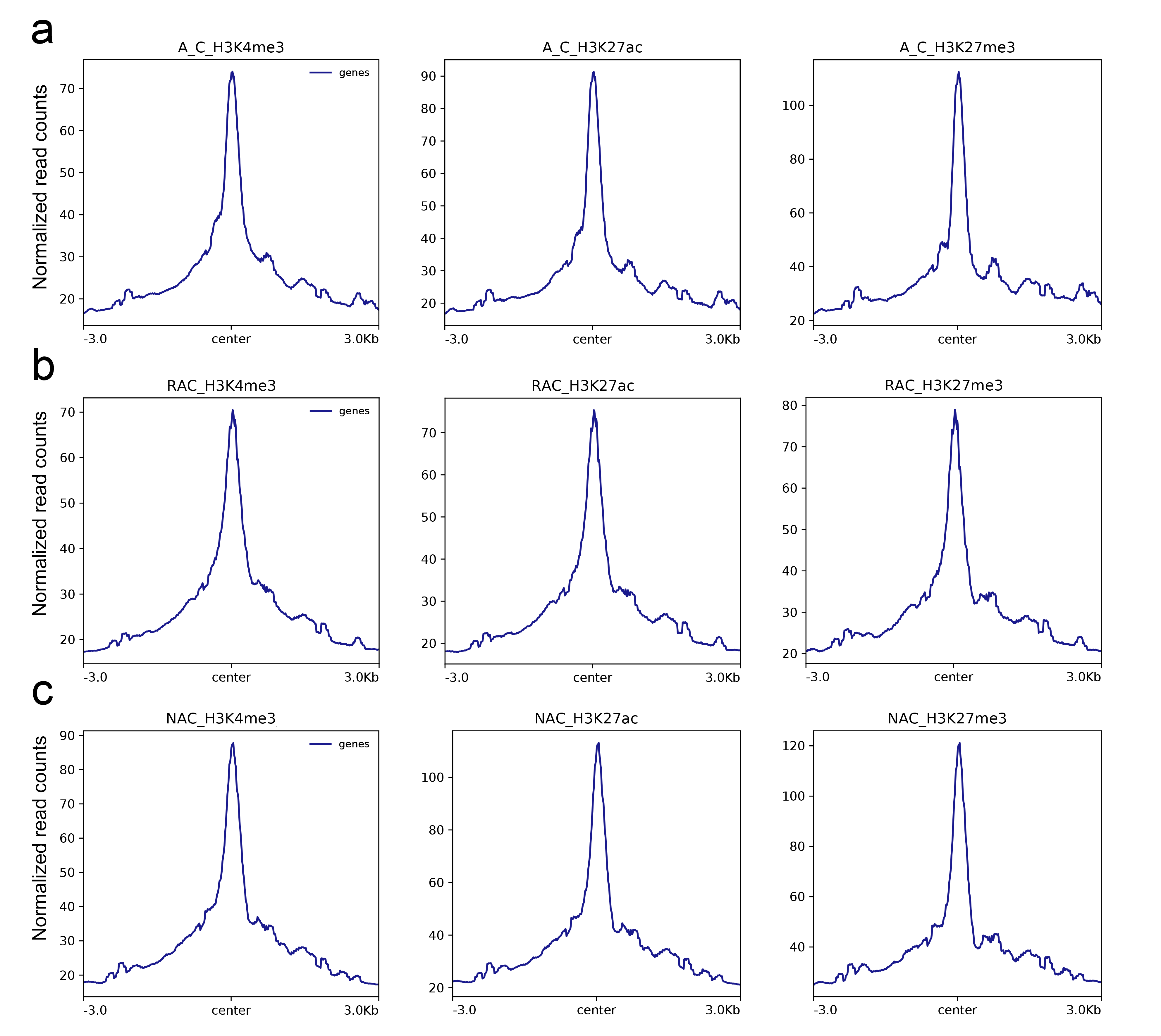
**

**Supplementary Fig. 7 Histone modifications around ACRs.** **a** H3K4me3, H3K27ac, and H3K27me3 around ACRs in A_C. **b** H3K4me3, H3K27ac, and H3K27me3 around ACRs in RAC. **c** H3K4me3, H3K27ac, and H3K27me3 around ACRs in NAC. A_C, *in silico* ‘hybrid’; RAC, resynthesized *B. napus*; NAC, natural *B. napus*; ACRs, accessible chromatin regions; center, peak center of ACRs.

**
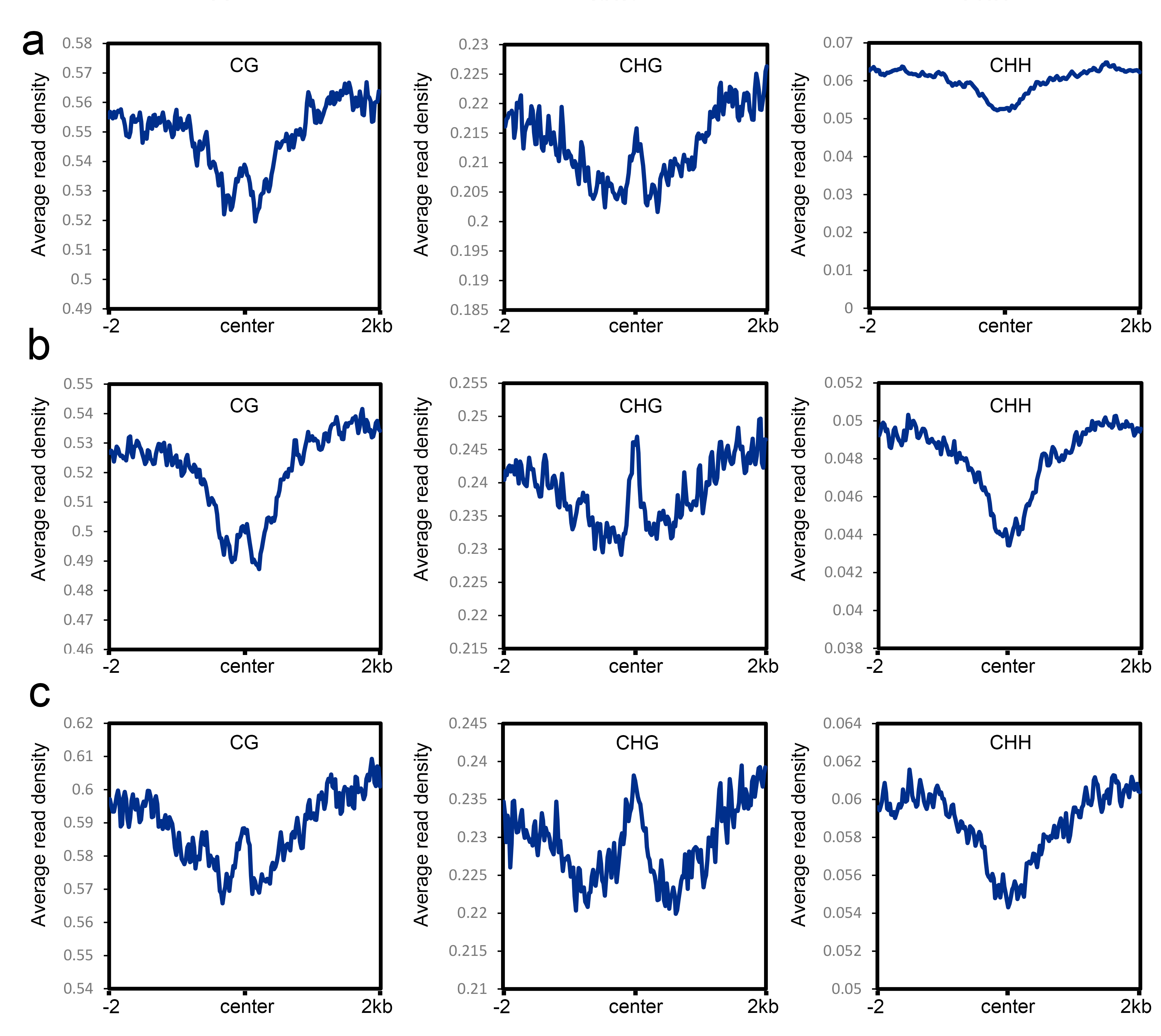
**

**Supplementary Fig. 8 DNA methylation around ACRs.** **a** CG, CHG, and CHH DNA methylation around ACRs in A_C. **b** CG, CHG, and CHH DNA methylation around ACRs in RAC. **c** CG, CHG, and CHH DNA methylation around ACRs in NAC. A_C, *in silico* ‘hybrid’; RAC, resynthesized *B. napus*; NAC, natural *B. napus*; ACRs, accessible chromatin regions; center, peak center of ACRs.

**
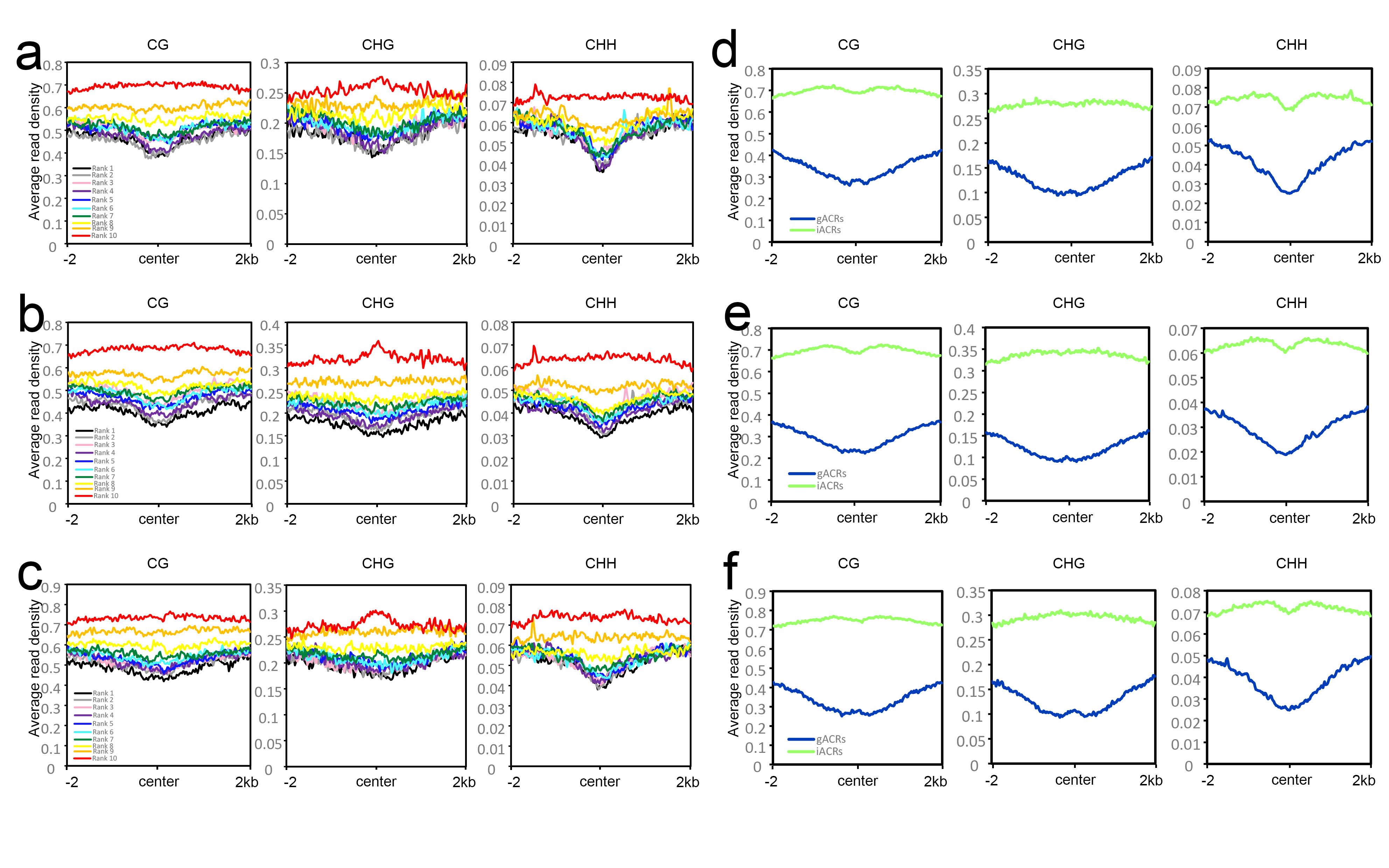
**

**Supplementary Fig. 9 DNA methylation around ACRs.** **a** CG, CHG, and CHH DNA methylation around ACRs with different chromatin accessibility in A_C. **b** CG, CHG, and CHH DNA methylation around ACRs with different chromatin accessibility in RAC. **c** CG, CHG, and CHH DNA methylation around ACRs with different chromatin accessibility in NAC. Values of 1 to 10 denote groups with low to high chromatin accessibility, respectively. **d** CG, CHG, and CHH DNA methylation around gACRs and iACRs in A_C. **e** CG, CHG, and CHH DNA methylation around gACRs and iACRs in RAC. **f** CG, CHG, and CHH DNA methylation around gACRs and iACRs in NAC. A_C, *in silico* ‘hybrid’; RAC, resynthesized *B. napus*; NAC, natural *B. napus*; ACRs, accessible chromatin regions; gACRs, genic ACRs; iACRs, intergenic ACRs; center, peak center of ACRs.


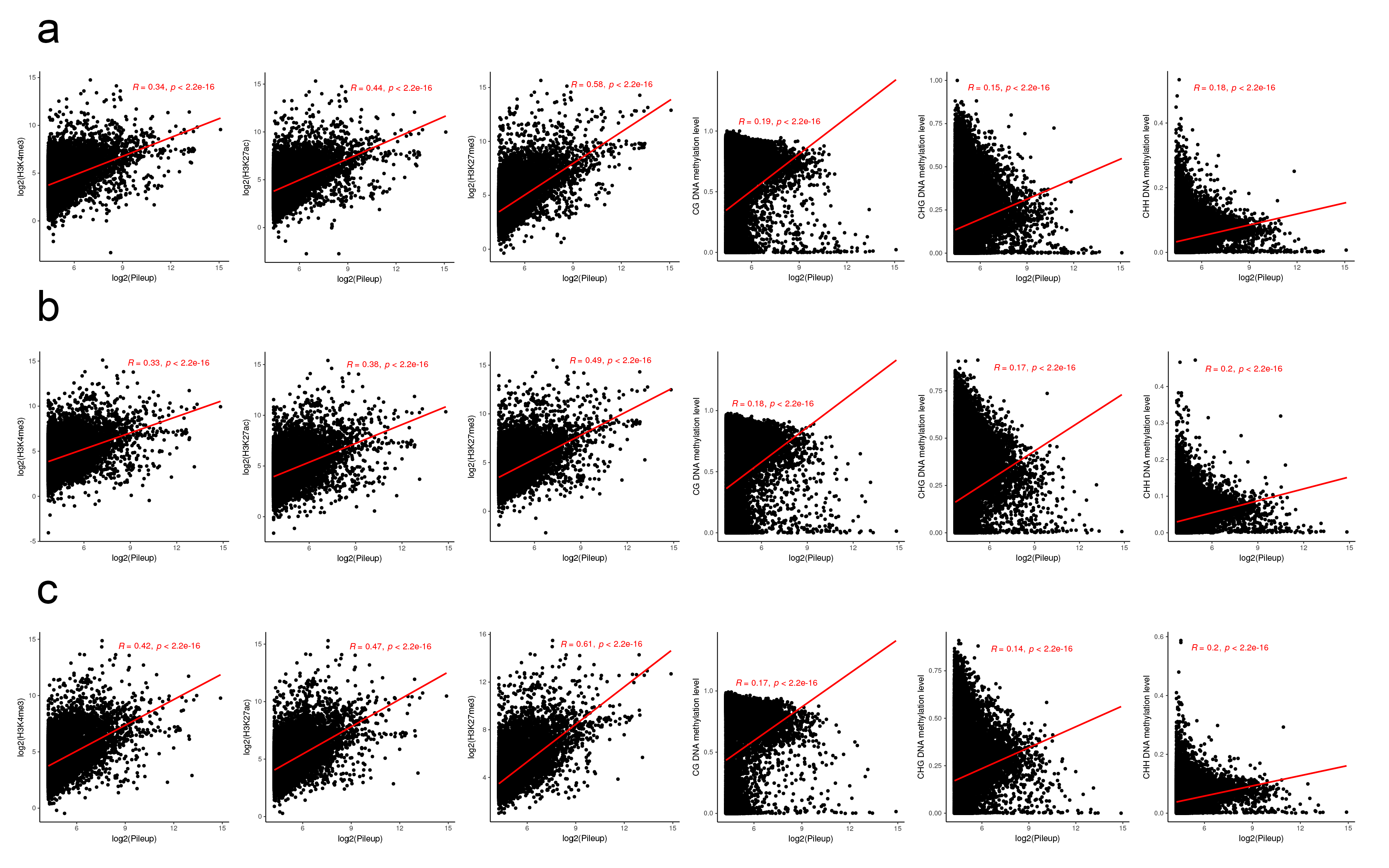


**Supplementary Fig. 10 Correlation analysis of intensity of ACRs with three histone modifications and DNA methylation.** **a** Correlation analysis of intensity of ACRs with H3K4me3, H3K27ac, H3K27me3 and DNA methylation in A_C. **b** Correlation analysis of intensity of ACRs with H3K4me3, H3K27ac, H3K27me3 and DNA methylation in RAC. **c** Correlation analysis of intensity of ACRs with H3K4me3, H3K27ac, H3K27me3 and DNA methylation in NAC. A_C, *in silico* ‘hybrid’; RAC, resynthesized *B. napus*; NAC, natural *B. napus*.

**
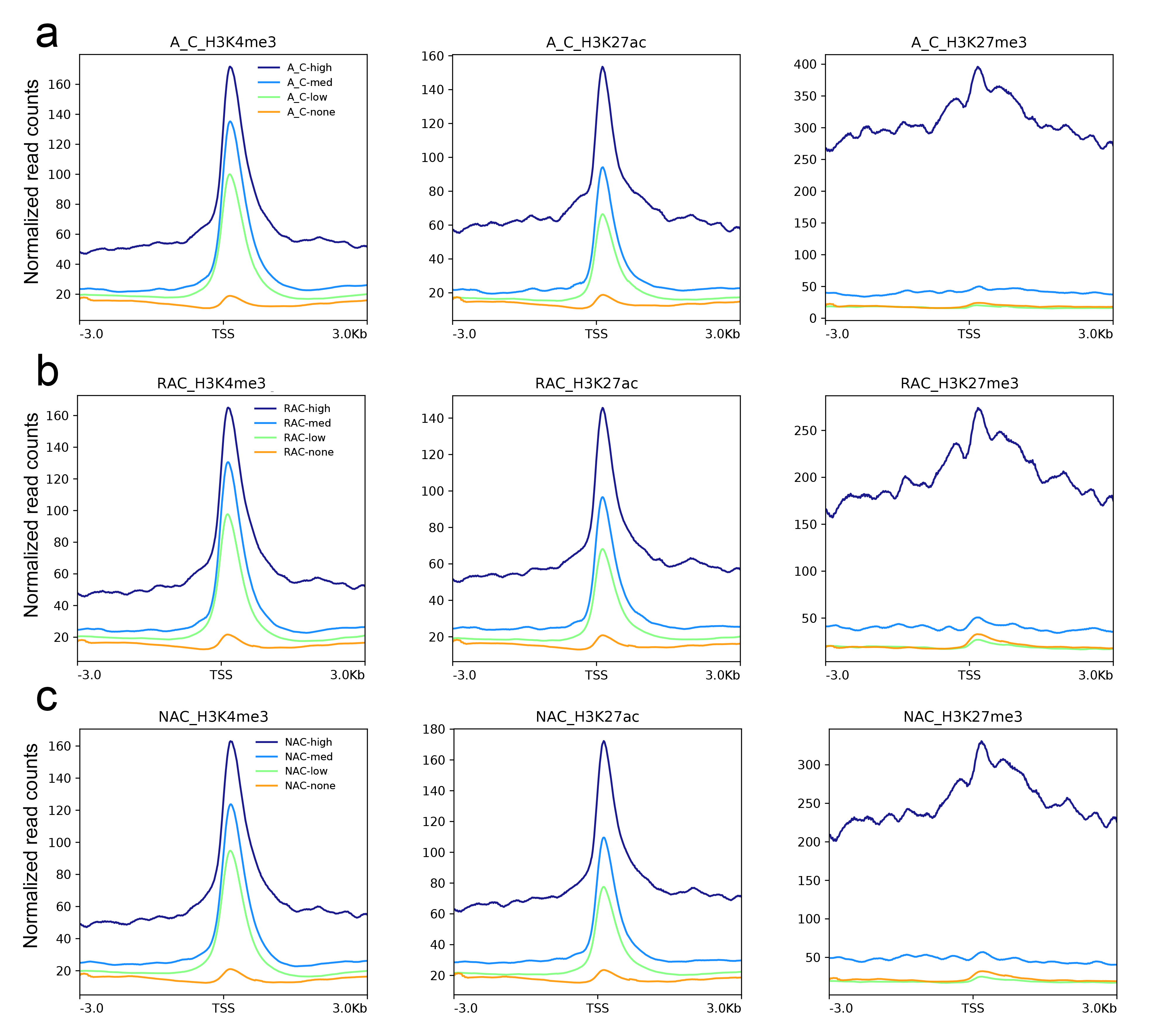
**

**Supplementary Fig. 11 The relationship between histone modifications and gene expression level.** **a** H3K4me3, H3K27ac, and H3K27me3 of genes with different expression levels in A_C. **b** H3K4me3, H3K27ac, and H3K27me3 of genes with different expression levels in RAC. **c** H3K4me3, H3K27ac, and H3K27me3 of genes with different expression levels in NAC. A_C, *in silico* ‘hybrid’; RAC, resynthesized *B. napus*; NAC, natural *B. napus*; TSS, transcript start site.

**
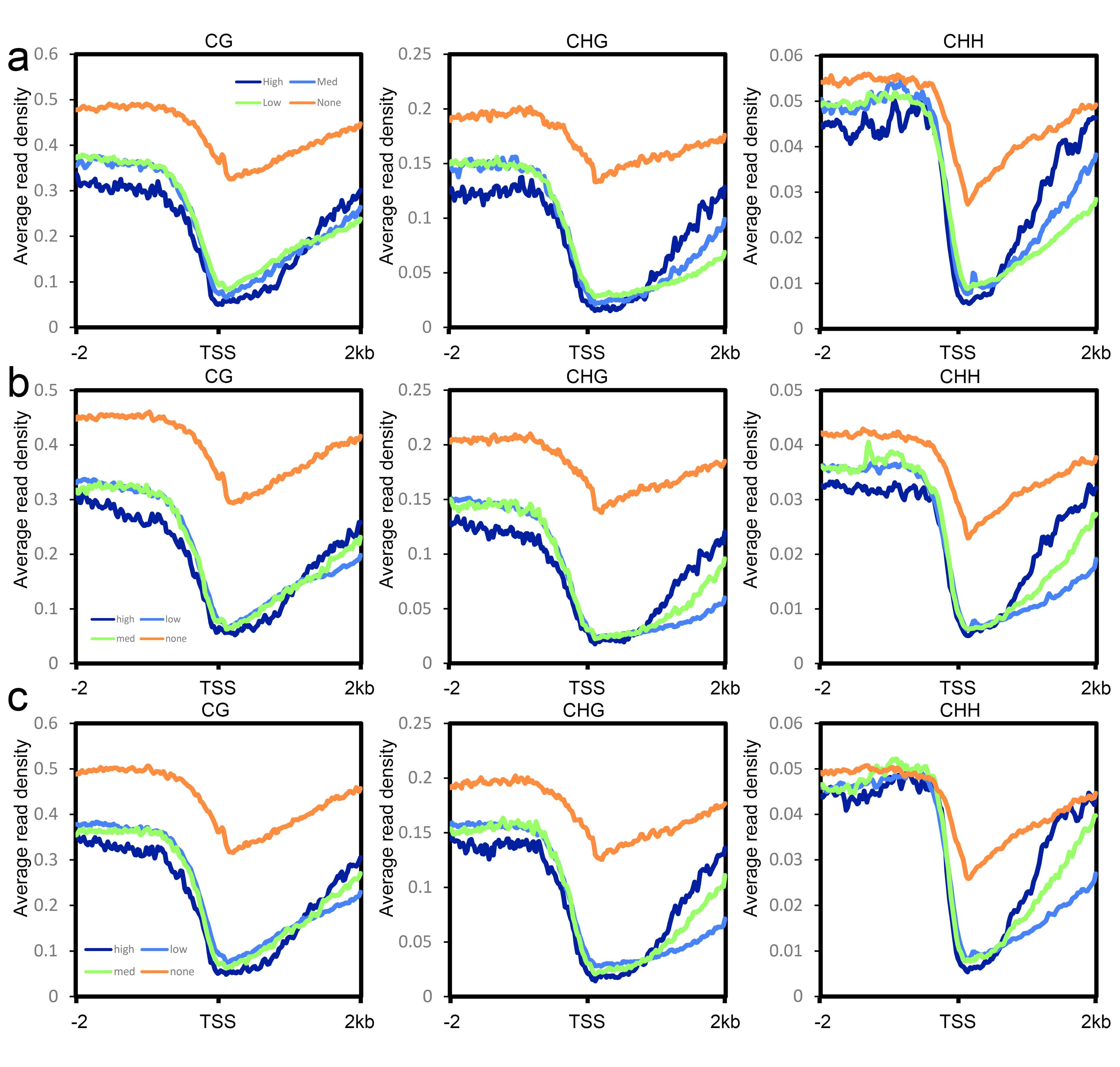
**

**Supplementary Fig. 12 The relationship between DNA methylation and gene expression level.** **a** CG, CHG, and CHH DNA methylation levels of genes with different expression levels in A_C. **b** CG, CHG, and CHH DNA methylation levels of genes with different expression levels in RAC. **c** CG, CHG, and CHH DNA methylation levels of genes with different expression levels in NAC. A_C, *in silico* ‘hybrid’; RAC, resynthesized *B. napus*; NAC, natural *B. napus*; TSS, transcript start site.

**
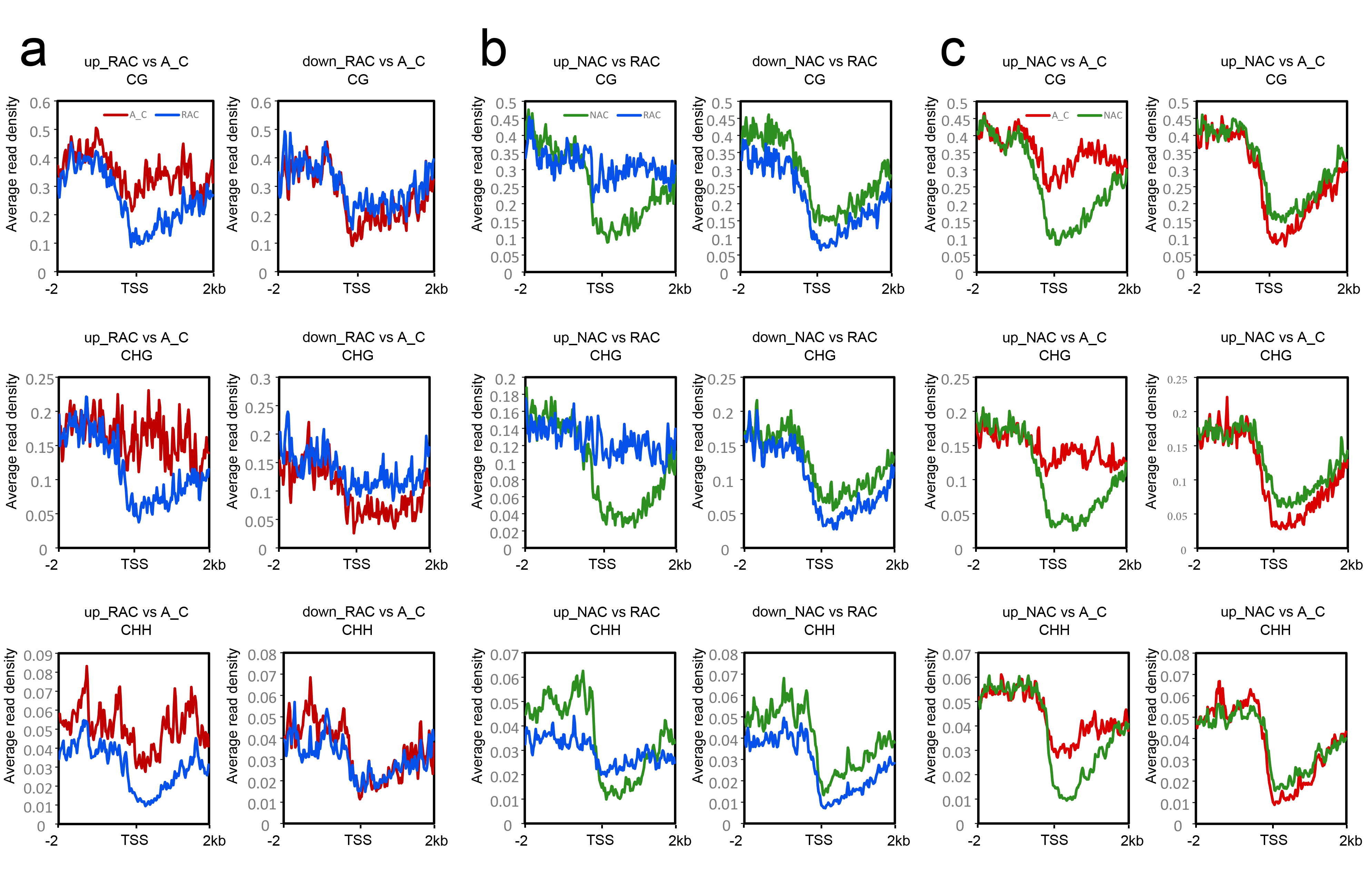
**

**Supplementary Fig. 13 The relationship between DEGs and DNA methylation.** **a** The relationship between DEGs and CG DNA methylation. **b** The relationship between DEGs and CHG DNA methylation. **c** The relationship between DEGs and CHH DNA methylation. DEGs, differential expressed genes; A_C, *in silico* ‘hybrid’; RAC, resynthesized *B. napus*; NAC, natural *B. napus*; TSS, transcriptional start site.

**
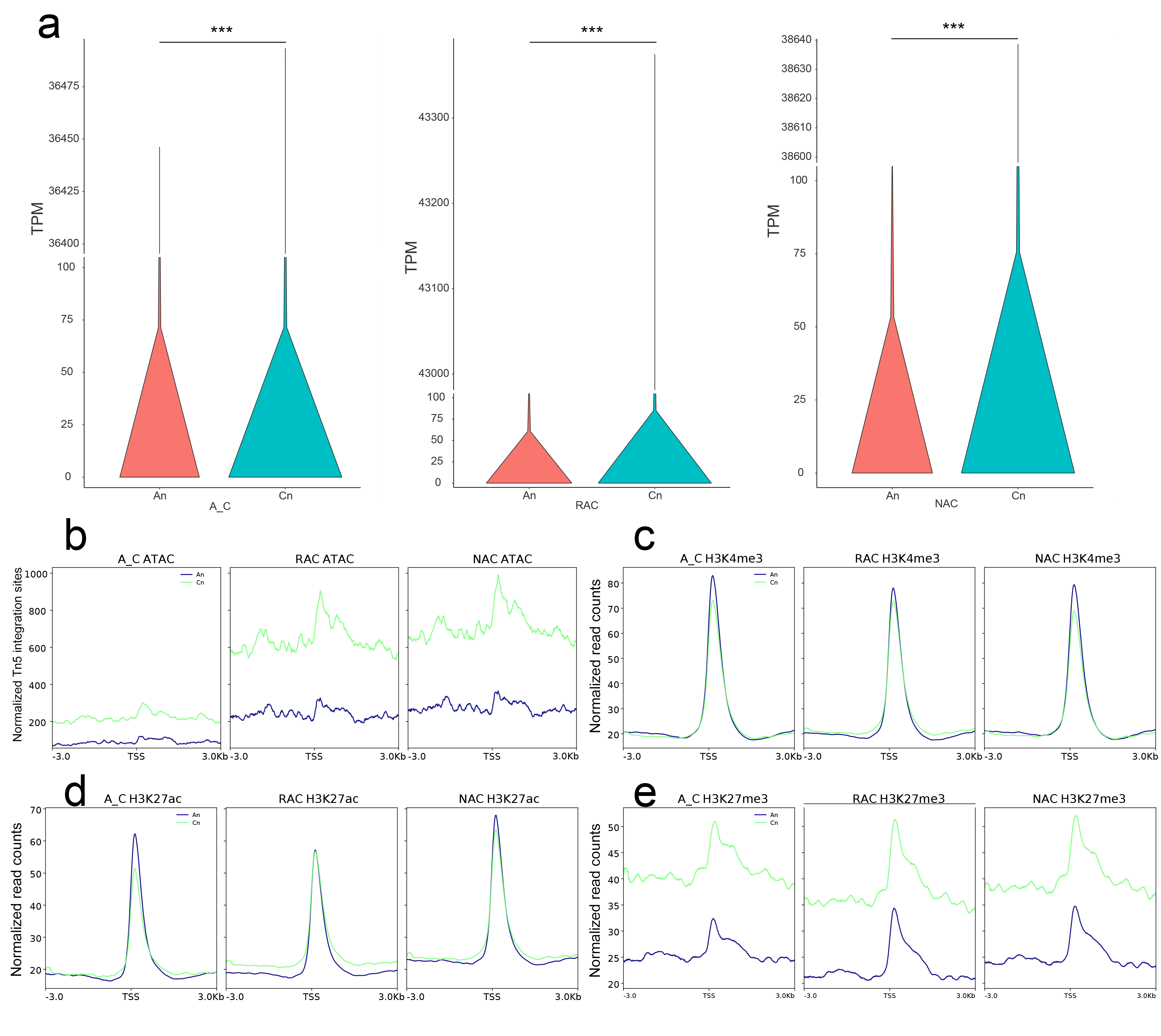
**

**Supplementary Fig. 14 Comparison of gene expression level and four epigenetic modifications between two subgenomes.** **a** Gene expression level of two subgenomes. **b** Chromatin accessibility of two subgenomes. **c** H3K4me3 level of two subgenomes. **d** H3K27ac level of two subgenomes. **e** H3K27me3 level of two subgenomes. A_C, *in silico* ‘hybrid’; RAC, resynthesized *B. napus*; NAC, natural *B. napus*; TSS, transcriptional start site.


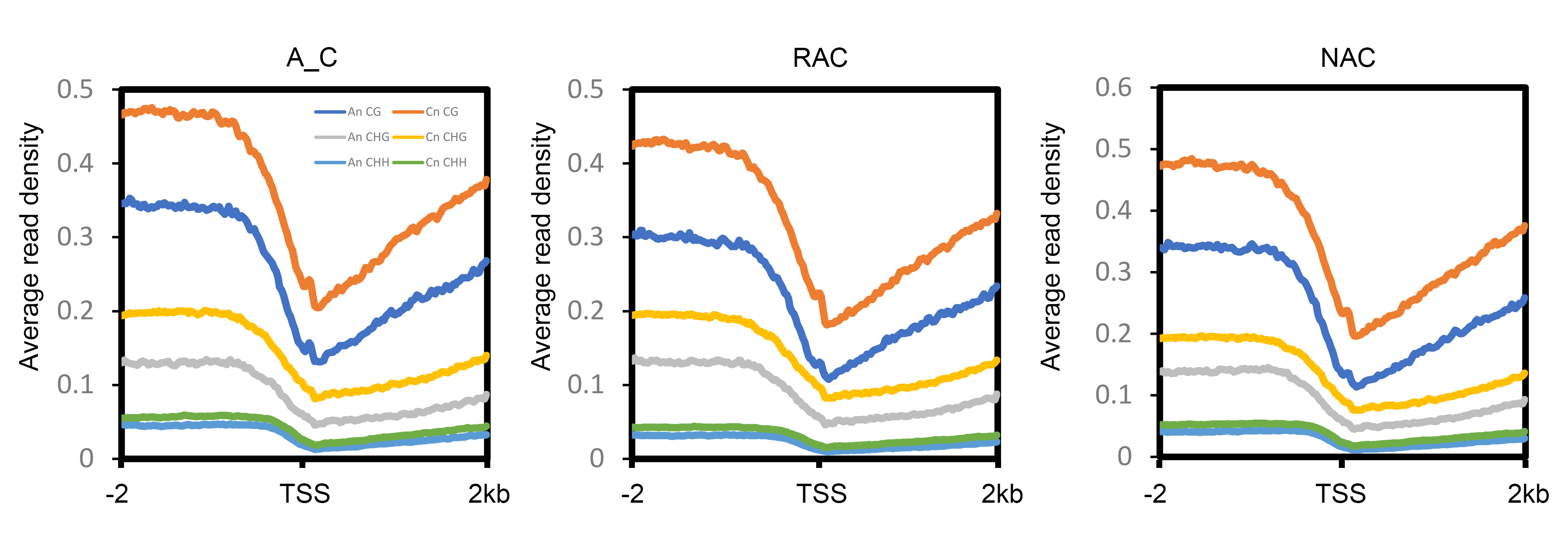


**Supplementary Fig. 15 Comparison of DNA methylation between two subgenomes.** A_C, *in silico* ‘hybrid’; RAC, resynthesized *B. napus*; NAC, natural *B. napus*; TSS, transcriptional start site.


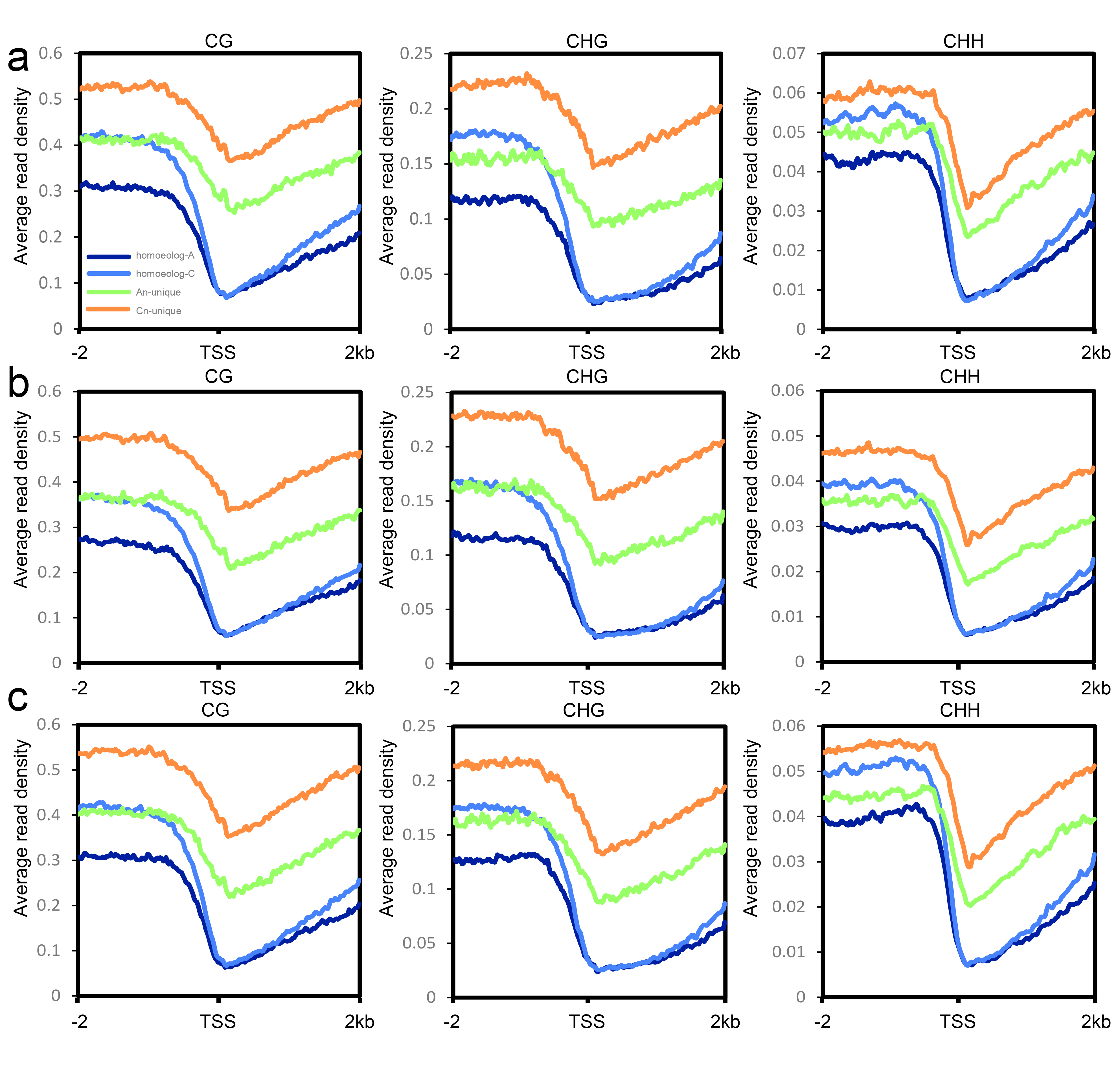


**Supplementary Fig. 16** **Comparison of three content of DNA methylation among homeologous genes and subgenome-unique genes in two subgenomes.** (a) DNA methylation level of homeologous genes and subgenome-unique genes in two subgenomes of A_C. (b) DNA methylation level of homeologous genes and subgenome-unique genes in two subgenomes of RAC. The color bar is the same as in (a). (c) DNA methylation level of homeologous genes and subgenome-unique genes in two subgenomes of NAC. The color bar is the same as in (a). A_C, *in silico* ‘hybrid’; RAC, resynthesized *B. napus*; NAC, natural *B. napus*; TSS, transcriptional start site.


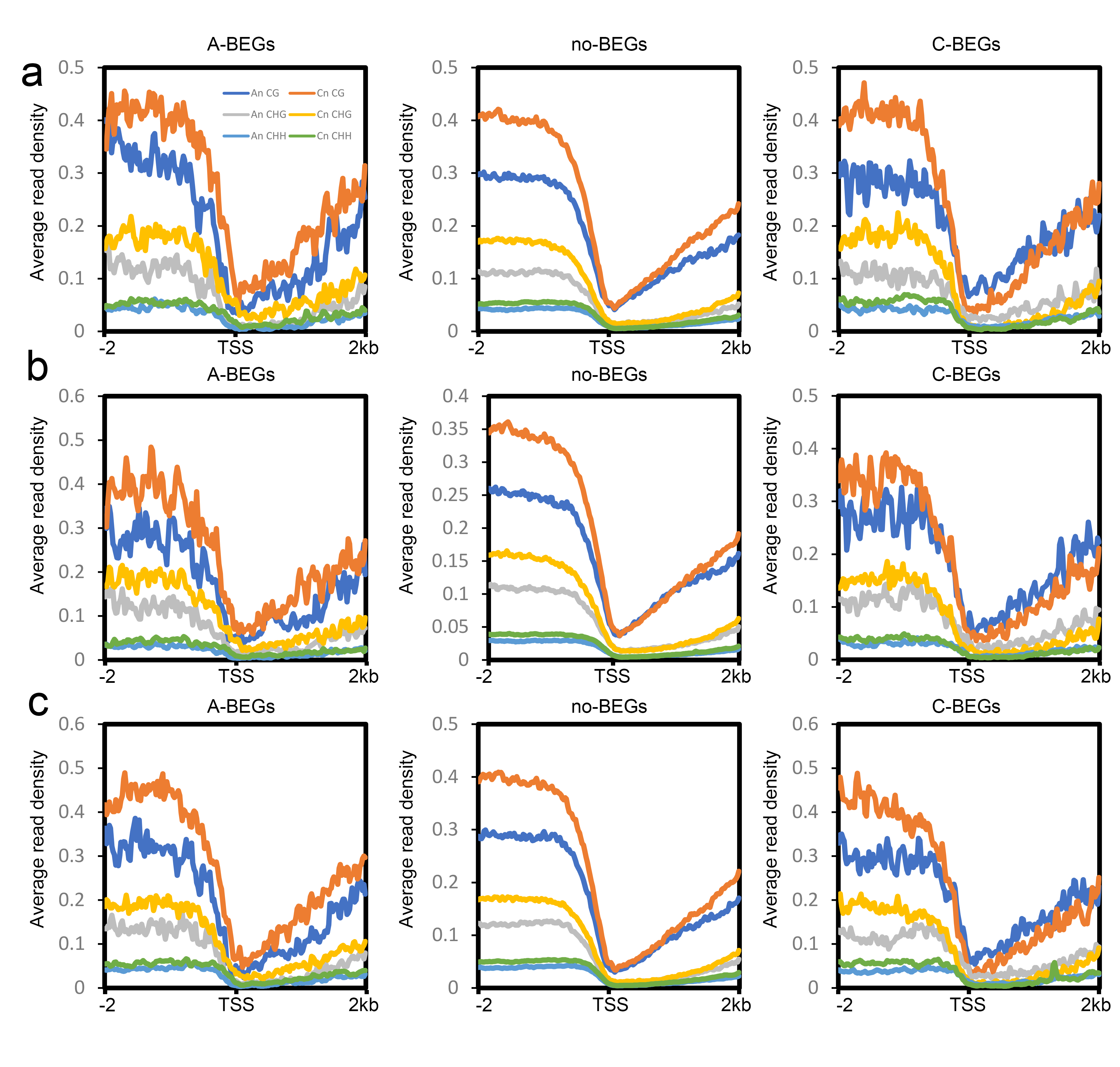


**Supplementary Fig. 17 Comparison of DNA methylation levels between homeologous genes pairs.** **a** DNA methylation levels of homeologous genes pairs in A_C. **b** DNA methylation levels of homeologous genes pairs in RAC. The color bar is the same as in (**a**). **c** DNA methylation levels of homeologous genes pairs in NAC. The color bar is the same as in (**a**). A_C, *in silico* ‘hybrid’; RAC, resynthesized *B. napus*; NAC, natural *B. napus*; TSS, transcriptional start site.

**Table S1** The preliminary analysis of ATAC-seq data for all samples

| Sample^a^ | Raw read | Clean read | Total mapped(%) | Q20(%) | Q30(%) | GC(%) |
| --- | --- | --- | --- | --- | --- | --- |
| A1 | 73,017,816 | 71,235,572 | 67,875,878(95.28) | 100 | 99.1 | 44 |
| A2 | 64,726,082 | 63,387,830 | 60,907,872(96.09) | 100 | 99.15 | 43 |
| A3 | 78,997,636 | 76,841,872 | 71,271,203(92.75) | 100 | 99.1 | 43 |
| C1 | 73,828,880 | 71,458,812 | 68,811,931(96.30) | 100 | 99.05 | 41 |
| C2 | 102,369,306 | 99,255,346 | 95,853,063(96.57) | 100 | 99.1 | 41 |
| C3 | 93,149,480 | 89,715,832 | 84,295,148(93.96) | 100 | 99.15 | 41 |
| RAC1 | 79,373,096 | 77,822,604 | 76,148,069(97.85) | 100 | 99.1 | 42 |
| RAC2 | 69,901,216 | 67,396,458 | 56,703,659(84.13) | 100 | 99.15 | 42 |
| RAC3 | 92,497,564 | 89,876,746 | 87,088,959(96.90) | 100 | 99.15 | 42.5 |
| NAC1 | 99,407,958 | 97,276,780 | 95,458,028(98.13) | 100 | 99.15 | 41 |
| NAC2 | 74,313,374 | 73,025,972 | 71,406,052(97.78) | 100 | 99.25 | 43 |
| NAC3 | 90,051,052 | 87,657,550 | 85,905,764(98.00) | 100 | 99.1 | 41 |

^a^1, 2 and 3 represented three biological replicates.

A, *B. rapa*; C, *B. oleracea*; RAC, resynthesized *B. napus*; NAC, natural *B. napus*.

**Table S2** Primers used in this study.

| Sample^a^ | Primer sequence (5' -> 3') | Name | Usage |
| --- | --- | --- | --- |
| A-H3K4me3-Input | AATGATACGGCGACCACCGAGATCTACACAGCGTAGCACACTCTTTCCCTACACGACGCTCTTCCGATCT | i5 index primers | Primers used to amplify ChIP-seq libraries |
| A-H3K4me3-Input | CAAGCAGAAGACGGCATACGAGATATTACTCGGTGACTGGAGTTCAGACGTGTGCT CTTCCGATCT | i7 index primers | Primers used to amplify ChIP-seq libraries |
| A-H3K4me3-IP | AATGATACGGCGACCACCGAGATCTACACAGCGTAGCACACTCTTTCCCTACACGACGCTCTTCCGATCT | i5 index primers | Primers used to amplify ChIP-seq libraries |
| A-H3K4me3-IP | CAAGCAGAAGACGGCATACGAGATTCCGGAGAGTGACTGGAGTTCAGACGTGTGCT CTTCCGATCT | i7 index primers | Primers used to amplify ChIP-seq libraries |
| A-H3K27ac-Input | AATGATACGGCGACCACCGAGATCTACACAGCGTAGCACACTCTTTCCCTACACGACGCTCTTCCGATCT | i5 index primers | Primers used to amplify ChIP-seq libraries |
| A-H3K27ac-Input | CAAGCAGAAGACGGCATACGAGATCGCTCATTGTGACTGGAGTTCAGACGTGTGCT CTTCCGATCT | i7 index primers | Primers used to amplify ChIP-seq libraries |
| A-H3K27ac-IP | AATGATACGGCGACCACCGAGATCTACACAGCGTAGCACACTCTTTCCCTACACGACGCTCTTCCGATCT | i5 index primers | Primers used to amplify ChIP-seq libraries |
| A-H3K27ac-IP | CAAGCAGAAGACGGCATACGAGATGAGATTCCGTGACTGGAGTTCAGACGTGTGCT CTTCCGATCT | i7 index primers | Primers used to amplify ChIP-seq libraries |
| A-H3K27me3-Input | AATGATACGGCGACCACCGAGATCTACACAGCGTAGCACACTCTTTCCCTACACGACGCTCTTCCGATCT | i5 index primers | Primers used to amplify ChIP-seq libraries |
| A-H3K27me3-Input | CAAGCAGAAGACGGCATACGAGATATTCAGAAGTGACTGGAGTTCAGACGTGTGCT CTTCCGATCT | i7 index primers | Primers used to amplify ChIP-seq libraries |
| A-H3K27me3-IP | AATGATACGGCGACCACCGAGATCTACACAGCGTAGCACACTCTTTCCCTACACGACGCTCTTCCGATCT | i5 index primers | Primers used to amplify ChIP-seq libraries |
| A-H3K27me3-IP | CAAGCAGAAGACGGCATACGAGATGAATTCGTGTGACTGGAGTTCAGACGTGTGCT CTTCCGATCT | i7 index primers | Primers used to amplify ChIP-seq libraries |
| NAC-H3K4me3-Input | AATGATACGGCGACCACCGAGATCTACACAGCGTAGCACACTCTTTCCCTACACGACGCTCTTCCGATCT | i5 index primers | Primers used to amplify ChIP-seq libraries |
| NAC-H3K4me3-Input | CAAGCAGAAGACGGCATACGAGATCTGAAGCTGTGACTGGAGTTCAGACGTGTGCT CTTCCGATCT | i7 index primers | Primers used to amplify ChIP-seq libraries |
| NAC-H3K4me3-IP | AATGATACGGCGACCACCGAGATCTACACAGCGTAGCACACTCTTTCCCTACACGACGCTCTTCCGATCT | i5 index primers | Primers used to amplify ChIP-seq libraries |
| NAC-H3K4me3-IP | CAAGCAGAAGACGGCATACGAGATTAATGCGCGTGACTGGAGTTCAGACGTGTGCT CTTCCGATCT | i7 index primers | Primers used to amplify ChIP-seq libraries |
| NAC-H3K27ac-Input | AATGATACGGCGACCACCGAGATCTACACAGCGTAGCACACTCTTTCCCTACACGACGCTCTTCCGATCT | i5 index primers | Primers used to amplify ChIP-seq libraries |
| NAC-H3K27ac-Input | CAAGCAGAAGACGGCATACGAGATCGGCTATGGTGACTGGAGTTCAGACGTGTGCT CTTCCGATCT | i7 index primers | Primers used to amplify ChIP-seq libraries |
| NAC-H3K27ac-IP | AATGATACGGCGACCACCGAGATCTACACAGCGTAGCACACTCTTTCCCTACACGACGCTCTTCCGATCT | i5 index primers | Primers used to amplify ChIP-seq libraries |
| NAC-H3K27ac-IP | CAAGCAGAAGACGGCATACGAGATTCCGCGAAGTGACTGGAGTTCAGACGTGTGCT CTTCCGATCT | i7 index primers | Primers used to amplify ChIP-seq libraries |
| NAC-H3K27me3-Input | AATGATACGGCGACCACCGAGATCTACACAGCGTAGCACACTCTTTCCCTACACGACGCTCTTCCGATCT | i5 index primers | Primers used to amplify ChIP-seq libraries |
| NAC-H3K27me3-Input | CAAGCAGAAGACGGCATACGAGATTCTCGCGCGTGACTGGAGTTCAGACGTGTGCT CTTCCGATCT | i7 index primers | Primers used to amplify ChIP-seq libraries |
| NAC-H3K27me3-IP | AATGATACGGCGACCACCGAGATCTACACAGCGTAGCACACTCTTTCCCTACACGACGCTCTTCCGATCT | i5 index primers | Primers used to amplify ChIP-seq libraries |
| NAC-H3K27me3-IP | CAAGCAGAAGACGGCATACGAGATAGCGATAGGTGACTGGAGTTCAGACGTGTGCT CTTCCGATCT | i7 index primers | Primers used to amplify ChIP-seq libraries |
| RAC-H3K4me3-Input | AATGATACGGCGACCACCGAGATCTACACTCCTCTACACACTCTTTCCCTACACGACGCTCTTCCGATCT | i5 index primers | Primers used to amplify ChIP-seq libraries |
| RAC-H3K4me3-Input | CAAGCAGAAGACGGCATACGAGATATTACTCGGTGACTGGAGTTCAGACGTGTGCT CTTCCGATCT | i7 index primers | Primers used to amplify ChIP-seq libraries |
| RAC-H3K4me3-IP | AATGATACGGCGACCACCGAGATCTACACTCCTCTACACACTCTTTCCCTACACGACGCTCTTCCGATCT | i5 index primers | Primers used to amplify ChIP-seq libraries |
| RAC-H3K4me3-IP | CAAGCAGAAGACGGCATACGAGATTCCGGAGAGTGACTGGAGTTCAGACGTGTGCT CTTCCGATCT | i7 index primers | Primers used to amplify ChIP-seq libraries |
| RAC-H3K27ac-Input | AATGATACGGCGACCACCGAGATCTACACTCCTCTACACACTCTTTCCCTACACGACGCTCTTCCGATCT | i5 index primers | Primers used to amplify ChIP-seq libraries |
| RAC-H3K27ac-Input | CAAGCAGAAGACGGCATACGAGATCGCTCATTGTGACTGGAGTTCAGACGTGTGCT CTTCCGATCT | i7 index primers | Primers used to amplify ChIP-seq libraries |
| RAC-H3K27ac-IP | AATGATACGGCGACCACCGAGATCTACACTCCTCTACACACTCTTTCCCTACACGACGCTCTTCCGATCT | i5 index primers | Primers used to amplify ChIP-seq libraries |
| RAC-H3K27ac-IP | CAAGCAGAAGACGGCATACGAGATGAGATTCCGTGACTGGAGTTCAGACGTGTGCT CTTCCGATCT | i7 index primers | Primers used to amplify ChIP-seq libraries |
| RAC-H3K27me3-Input | AATGATACGGCGACCACCGAGATCTACACTCCTCTACACACTCTTTCCCTACACGACGCTCTTCCGATCT | i5 index primers | Primers used to amplify ChIP-seq libraries |
| RAC-H3K27me3-Input | CAAGCAGAAGACGGCATACGAGATATTCAGAAGTGACTGGAGTTCAGACGTGTGCT CTTCCGATCT | i7 index primers | Primers used to amplify ChIP-seq libraries |
| RAC-H3K27me3-IP | AATGATACGGCGACCACCGAGATCTACACTCCTCTACACACTCTTTCCCTACACGACGCTCTTCCGATCT | i5 index primers | Primers used to amplify ChIP-seq libraries |
| RAC-H3K27me3-IP | CAAGCAGAAGACGGCATACGAGATGAATTCGTGTGACTGGAGTTCAGACGTGTGCT CTTCCGATCT | i7 index primers | Primers used to amplify ChIP-seq libraries |
| C-H3K4me3-Input | AATGATACGGCGACCACCGAGATCTACACTCCTCTACACACTCTTTCCCTACACGACGCTCTTCCGATCT | i5 index primers | Primers used to amplify ChIP-seq libraries |
| C-H3K4me3-Input | CAAGCAGAAGACGGCATACGAGATCTGAAGCTGTGACTGGAGTTCAGACGTGTGCT CTTCCGATCT | i7 index primers | Primers used to amplify ChIP-seq libraries |
| C-H3K4me3-IP | AATGATACGGCGACCACCGAGATCTACACTCCTCTACACACTCTTTCCCTACACGACGCTCTTCCGATCT | i5 index primers | Primers used to amplify ChIP-seq libraries |
| C-H3K4me3-IP | CAAGCAGAAGACGGCATACGAGATTAATGCGCGTGACTGGAGTTCAGACGTGTGCT CTTCCGATCT | i7 index primers | Primers used to amplify ChIP-seq libraries |
| C-H3K27ac-Input | AATGATACGGCGACCACCGAGATCTACACTCCTCTACACACTCTTTCCCTACACGACGCTCTTCCGATCT | i5 index primers | Primers used to amplify ChIP-seq libraries |
| C-H3K27ac-Input | CAAGCAGAAGACGGCATACGAGATCGGCTATGGTGACTGGAGTTCAGACGTGTGCT CTTCCGATCT | i7 index primers | Primers used to amplify ChIP-seq libraries |
| C-H3K27ac-IP | AATGATACGGCGACCACCGAGATCTACACTCCTCTACACACTCTTTCCCTACACGACGCTCTTCCGATCT | i5 index primers | Primers used to amplify ChIP-seq libraries |
| C-H3K27ac-IP | CAAGCAGAAGACGGCATACGAGATTCCGCGAAGTGACTGGAGTTCAGACGTGTGCT CTTCCGATCT | i7 index primers | Primers used to amplify ChIP-seq libraries |
| C-H3K27me3-Input | AATGATACGGCGACCACCGAGATCTACACTCCTCTACACACTCTTTCCCTACACGACGCTCTTCCGATCT | i5 index primers | Primers used to amplify ChIP-seq libraries |
| C-H3K27me3-Input | CAAGCAGAAGACGGCATACGAGATTCTCGCGCGTGACTGGAGTTCAGACGTGTGCT CTTCCGATCT | i7 index primers | Primers used to amplify ChIP-seq libraries |
| C-H3K27me3-IP | AATGATACGGCGACCACCGAGATCTACACTCCTCTACACACTCTTTCCCTACACGACGCTCTTCCGATCT | i5 index primers | Primers used to amplify ChIP-seq libraries |
| C-H3K27me3-IP | CAAGCAGAAGACGGCATACGAGATAGCGATAGGTGACTGGAGTTCAGACGTGTGCT CTTCCGATCT | i7 index primers | Primers used to amplify ChIP-seq libraries |
| A1 | AATGATACGGCGACCACCGAGATCTACACACTGCATAACACTCTTTCCCTACACGACGCTCTTCCGATCT | i5 index primers | Primers used to amplify ATAC-seq libraries |
| A1 | CAAGCAGAAGACGGCATACGAGATCTCTCTACGTGACTGGAGTTCAGACGTGTGCT CTTCCGATCT | i7 index primers | Primers used to amplify ATAC-seq libraries |
| A2 | AATGATACGGCGACCACCGAGATCTACACACTGCATAACACTCTTTCCCTACACGACGCTCTTCCGATCT | i5 index primers | Primers used to amplify ATAC-seq libraries |
| A2 | CAAGCAGAAGACGGCATACGAGATCAGAGAGGGTGACTGGAGTTCAGACGTGTGCT CTTCCGATCT | i7 index primers | Primers used to amplify ATAC-seq libraries |
| A3 | AATGATACGGCGACCACCGAGATCTACACACTGCATAACACTCTTTCCCTACACGACGCTCTTCCGATCT | i5 index primers | Primers used to amplify ATAC-seq libraries |
| A3 | CAAGCAGAAGACGGCATACGAGATGCTACGCTGTGACTGGAGTTCAGACGTGTGCT CTTCCGATCT | i7 index primers | Primers used to amplify ATAC-seq libraries |
| C1 | AATGATACGGCGACCACCGAGATCTACACACTGCATAACACTCTTTCCCTACACGACGCTCTTCCGATCT | i5 index primers | Primers used to amplify ATAC-seq libraries |
| C1 | CAAGCAGAAGACGGCATACGAGATAGGCAGAAGTGACTGGAGTTCAGACGTGTGCT CTTCCGATCT | i7 index primers | Primers used to amplify ATAC-seq libraries |
| C2 | AATGATACGGCGACCACCGAGATCTACACACTGCATAACACTCTTTCCCTACACGACGCTCTTCCGATCT | i5 index primers | Primers used to amplify ATAC-seq libraries |
| C2 | CAAGCAGAAGACGGCATACGAGATGGACTCCTGTGACTGGAGTTCAGACGTGTGCT CTTCCGATCT | i7 index primers | Primers used to amplify ATAC-seq libraries |
| C3 | AATGATACGGCGACCACCGAGATCTACACACTGCATAACACTCTTTCCCTACACGACGCTCTTCCGATCT | i5 index primers | Primers used to amplify ATAC-seq libraries |
| C3 | CAAGCAGAAGACGGCATACGAGATTAGGCATGGTGACTGGAGTTCAGACGTGTGCT CTTCCGATCT | i7 index primers | Primers used to amplify ATAC-seq libraries |
| NAC-1 | AATGATACGGCGACCACCGAGATCTACACGTAAGGAGACACTCTTTCCCTACACGACGCTCTTCCGATCT | i5 index primers | Primers used to amplify ATAC-seq libraries |
| NAC-1 | CAAGCAGAAGACGGCATACGAGATCTCTCTACGTGACTGGAGTTCAGACGTGTGCT CTTCCGATCT | i7 index primers | Primers used to amplify ATAC-seq libraries |
| NAC-2 | AATGATACGGCGACCACCGAGATCTACACGTAAGGAGACACTCTTTCCCTACACGACGCTCTTCCGATCT | i5 index primers | Primers used to amplify ATAC-seq libraries |
| NAC-2 | CAAGCAGAAGACGGCATACGAGATCAGAGAGGGTGACTGGAGTTCAGACGTGTGCT CTTCCGATCT | i7 index primers | Primers used to amplify ATAC-seq libraries |
| NAC-3 | AATGATACGGCGACCACCGAGATCTACACGTAAGGAGACACTCTTTCCCTACACGACGCTCTTCCGATCT | i5 index primers | Primers used to amplify ATAC-seq libraries |
| NAC-3 | CAAGCAGAAGACGGCATACGAGATGCTACGCTGTGACTGGAGTTCAGACGTGTGCT CTTCCGATCT | i7 index primers | Primers used to amplify ATAC-seq libraries |
| RAC-1 | AATGATACGGCGACCACCGAGATCTACACGTAAGGAGACACTCTTTCCCTACACGACGCTCTTCCGATCT | i5 index primers | Primers used to amplify ATAC-seq libraries |
| RAC-1 | CAAGCAGAAGACGGCATACGAGATAGGCAGAAGTGACTGGAGTTCAGACGTGTGCT CTTCCGATCT | i7 index primers | Primers used to amplify ATAC-seq libraries |
| RAC-2 | AATGATACGGCGACCACCGAGATCTACACGTAAGGAGACACTCTTTCCCTACACGACGCTCTTCCGATCT | i5 index primers | Primers used to amplify ATAC-seq libraries |
| RAC-2 | CAAGCAGAAGACGGCATACGAGATGGACTCCTGTGACTGGAGTTCAGACGTGTGCT CTTCCGATCT | i7 index primers | Primers used to amplify ATAC-seq libraries |
| RAC-3 | AATGATACGGCGACCACCGAGATCTACACGTAAGGAGACACTCTTTCCCTACACGACGCTCTTCCGATCT | i5 index primers | Primers used to amplify ATAC-seq libraries |
| RAC-3 | CAAGCAGAAGACGGCATACGAGATTAGGCATGGTGACTGGAGTTCAGACGTGTGCT CTTCCGATCT | i7 index primers | Primers used to amplify ATAC-seq libraries |
| A1 | AATGATACGGCGACCACCGAGATCTACACCGTCTAATTCGTCGGCAGCGTCAGATGTGTATAAGAGACAG | i5 index primers | Primers used to amplify WGBS libraries |
| A1 | CTGTCTCTTATACACATCTCCGAGCCCACGAGACCATTGCTTATCTCGTATGCCGTCTTCTGCTTG | i7 index primers | Primers used to amplify WGBS libraries |
| A2 | AATGATACGGCGACCACCGAGATCTACACCGTCTAATTCGTCGGCAGCGTCAGATGTGTATAAGAGACAG | i5 index primers | Primers used to amplify WGBS libraries |
| A2 | CTGTCTCTTATACACATCTCCGAGCCCACGAGACTTCGGATTATCTCGTATGCCGTCTTCTGCTTG | i7 index primers | Primers used to amplify WGBS libraries |
| A3 | AATGATACGGCGACCACCGAGATCTACACCGTCTAATTCGTCGGCAGCGTCAGATGTGTATAAGAGACAG | i5 index primers | Primers used to amplify WGBS libraries |
| A3 | CTGTCTCTTATACACATCTCCGAGCCCACGAGACTCATCATTATCTCGTATGCCGTCTTCTGCTTG | i7 index primers | Primers used to amplify WGBS libraries |
| C1 | AATGATACGGCGACCACCGAGATCTACACCGTCTAATTCGTCGGCAGCGTCAGATGTGTATAAGAGACAG | i5 index primers | Primers used to amplify WGBS libraries |
| C1 | CTGTCTCTTATACACATCTCCGAGCCCACGAGACGCTCCTGTATCTCGTATGCCGTCTTCTGCTTG | i7 index primers | Primers used to amplify WGBS libraries |
| C2 | AATGATACGGCGACCACCGAGATCTACACCGTCTAATTCGTCGGCAGCGTCAGATGTGTATAAGAGACAG | i5 index primers | Primers used to amplify WGBS libraries |
| C2 | CTGTCTCTTATACACATCTCCGAGCCCACGAGACAGCTCGGTATCTCGTATGCCGTCTTCTGCTTG | i7 index primers | Primers used to amplify WGBS libraries |
| C3 | AATGATACGGCGACCACCGAGATCTACACCGTCTAATTCGTCGGCAGCGTCAGATGTGTATAAGAGACAG | i5 index primers | Primers used to amplify WGBS libraries |
| C3 | CTGTCTCTTATACACATCTCCGAGCCCACGAGACCAACAGGTATCTCGTATGCCGTCTTCTGCTTG | i7 index primers | Primers used to amplify WGBS libraries |
| NAC1 | AATGATACGGCGACCACCGAGATCTACACCGTCTAATTCGTCGGCAGCGTCAGATGTGTATAAGAGACAG | i5 index primers | Primers used to amplify WGBS libraries |
| NAC1 | CTGTCTCTTATACACATCTCCGAGCCCACGAGACGTAAGAGTATCTCGTATGCCGTCTTCTGCTTG | i7 index primers | Primers used to amplify WGBS libraries |
| NAC2 | AATGATACGGCGACCACCGAGATCTACACCGTCTAATTCGTCGGCAGCGTCAGATGTGTATAAGAGACAG | i5 index primers | Primers used to amplify WGBS libraries |
| NAC2 | CTGTCTCTTATACACATCTCCGAGCCCACGAGACAAGTCTCTATCTCGTATGCCGTCTTCTGCTTG | i7 index primers | Primers used to amplify WGBS libraries |
| NAC3 | AATGATACGGCGACCACCGAGATCTACACCGTCTAATTCGTCGGCAGCGTCAGATGTGTATAAGAGACAG | i5 index primers | Primers used to amplify WGBS libraries |
| NAC3 | CTGTCTCTTATACACATCTCCGAGCCCACGAGACAGAGATCTATCTCGTATGCCGTCTTCTGCTTG | i7 index primers | Primers used to amplify WGBS libraries |
| RAC1 | AATGATACGGCGACCACCGAGATCTACACCGTCTAATTCGTCGGCAGCGTCAGATGTGTATAAGAGACAG | i5 index primers | Primers used to amplify WGBS libraries |
| RAC1 | CTGTCTCTTATACACATCTCCGAGCCCACGAGACTTCAAGGTATCTCGTATGCCGTCTTCTGCTTG | i7 index primers | Primers used to amplify WGBS libraries |
| RAC2 | AATGATACGGCGACCACCGAGATCTACACCGTCTAATTCGTCGGCAGCGTCAGATGTGTATAAGAGACAG | i5 index primers | Primers used to amplify WGBS libraries |
| RAC2 | CTGTCTCTTATACACATCTCCGAGCCCACGAGACCCTAACGTATCTCGTATGCCGTCTTCTGCTTG | i7 index primers | Primers used to amplify WGBS libraries |
| RAC3 | AATGATACGGCGACCACCGAGATCTACACCGTCTAATTCGTCGGCAGCGTCAGATGTGTATAAGAGACAG | i5 index primers | Primers used to amplify WGBS libraries |
| RAC3 | CTGTCTCTTATACACATCTCCGAGCCCACGAGACCACGTAGTATCTCGTATGCCGTCTTCTGCTTG | i7 index primers | Primers used to amplify WGBS libraries |

^a^1, 2 and 3 represented three biological replicates.

A, *B. rapa*; C, *B. oleracea*; RAC, resynthesized *B. napus*; NAC, natural *B. napus*.
