# Supplementary material for: Asymmetric subgenomic chromatin architecture impacts on gene expression in resynthesized and natural allopolyploid Brassica napus
Source: Commun Biol. 2022 Jul 29;5:762. doi: 10.1038/s42003-022-03729-7 (PMC9338098; doi:10.1038/s42003-022-03729-7)
Supplement: Supplementary file 3 — Description of Additional Supplementary Files [file 42003_2022_3729_MOESM3_ESM.pdf]

Description of Additional Supplementary Files

File name: Supplementary Data 1
Description: GO enrichment analysis of ACRs-associated genes in the three genotypes.

File name: Supplementary Data 2
Description: GO enrichment analysis of genes associated with different enriched ACRs.

File name: Supplementary Data 3
Description: Overrepresented motifs and corresponding TF families.

File name: Supplementary Data 4
Description: GO enrichment analysis of common and genotype-specific ACRs-associated genes in the three genotypes.

File name: Supplementary Data 5
Description: Source data underlying the graphs and charts presented in the main figures.
