# Supplementary material for: Asymmetric subgenomic chromatin architecture impacts on gene expression in resynthesized and natural allopolyploid Brassica napus
Source: Commun Biol. 2022 Jul 29;5:762. doi: 10.1038/s42003-022-03729-7 (PMC9338098; doi:10.1038/s42003-022-03729-7)
Supplement: Supplementary file 9 — Reporting Summary [file 42003_2022_3729_MOESM9_ESM.pdf]

nature portfolio

Corresponding author(s): Jianbo Wang

Last updated by author(s): May 27, 2022

Reporting Summary

Nature Portfolio wishes to improve the reproducibility of the work that we publish. This form provides structure for consistency and transparency in reporting. For further information on Nature Portfolio policies, see our Editorial Policies and the Editorial Policy Checklist.

Statistics

For all statistical analyses, confirm that the following items are present in the figure legend, table legend, main text, or Methods section.

n/a	Confirmed	
☐	☒	The exact sample size (*n*) for each experimental group/condition, given as a discrete number and unit of measurement
☐	☒	A statement on whether measurements were taken from distinct samples or whether the same sample was measured repeatedly
☐	☒	The statistical test(s) used AND whether they are one- or two-sided<br>*Only common tests should be described solely by name; describe more complex techniques in the Methods section.*
☒	☐	A description of all covariates tested
☒	☐	A description of any assumptions or corrections, such as tests of normality and adjustment for multiple comparisons
☐	☒	A full description of the statistical parameters including central tendency (e.g. means) or other basic estimates (e.g. regression coefficient) AND variation (e.g. standard deviation) or associated estimates of uncertainty (e.g. confidence intervals)
☒	☐	For null hypothesis testing, the test statistic (e.g. *F*, *t*, *r*) with confidence intervals, effect sizes, degrees of freedom and *P* value noted<br>*Give P values as exact values whenever suitable.*
☒	☐	For Bayesian analysis, information on the choice of priors and Markov chain Monte Carlo settings
☒	☐	For hierarchical and complex designs, identification of the appropriate level for tests and full reporting of outcomes
☒	☐	Estimates of effect sizes (e.g. Cohen's *d*, Pearson's *r*), indicating how they were calculated

Our web collection on statistics for biologists contains articles on many of the points above.

Software and code

Policy information about availability of computer code

Data collection	No software was used.
Data analysis	FastQC, Trimmomatic, STAR, MACS2, bedtools, deeptools, FastUniq, edgeR, bowtie2, samtools, HOMER, ChIPseeker, NanoFilt, Pinfish, DESeq2, KOBAS, BatMeth2.

For manuscripts utilizing custom algorithms or software that are central to the research but not yet described in published literature, software must be made available to editors and reviewers. We strongly encourage code deposition in a community repository (e.g. GitHub). See the Nature Portfolio guidelines for submitting code & software for further information.

Data

Policy information about availability of data

All manuscripts must include a data availability statement. This statement should provide the following information, where applicable:

- Accession codes, unique identifiers, or web links for publicly available datasets
- A description of any restrictions on data availability
- For clinical datasets or third party data, please ensure that the statement adheres to our policy

The data of ATAC-Seq, RNA-Seq, ChIP-Seq, and DNA methylation are available in the National Center for Biotechnology Information (NCBI) Sequence Read Archive (SRA) with the accession numbers SRR17818023-SRR17818034, SRR13302173-SRR13302184, SRR13318007-SRR13318030, and SRR13306925-SRR13306936, respectively.

Human research participants

Policy information about studies involving human research participants and Sex and Gender in Research.

Reporting on sex and gender	This is not applicable in our study.
Population characteristics	This is not applicable in our study.
Recruitment	This is not applicable in our study.
Ethics oversight	This is not applicable in our study.

Note that full information on the approval of the study protocol must also be provided in the manuscript.

Field-specific reporting

Please select the one below that is the best fit for your research. If you are not sure, read the appropriate sections before making your selection.

☐ Life sciences ☐ Behavioural & social sciences ☒ Ecological, evolutionary & environmental sciences

For a reference copy of the document with all sections, see nature.com/documents/nr-reporting-summary-flat.pdf

Ecological, evolutionary & environmental sciences study design

All studies must disclose on these points even when the disclosure is negative.

Study description	In this study, we comprehensively analyzed genome-wide chromatin accessibility of natural B. napus (natural establishment experienced about 7500 years), resynthesized B. napus (the newly formation) and their progenitors using ATAC-Seq. In addition, we systematically analyzed the potential relationship between chromatin accessibility and gene expression and four epigenetic modification (H3K4me3, H3K27me3, H3K27ac, and DNA methylation) during the newly formation and natural evolution process of B. napus.
Research sample	Natural allotetraploid B. napus L. (cv. Darmor), resynthesized allotetraploid B. napus L. (HC-2) and its parents B. rapa L. (cv. 9JC002, paternal), B. oleracea L. (cv. 3YS013, maternal).
Sampling strategy	Through pre-experiment, it was determined that leaves of five-week old plants were enough to meet the requirements of sample size for database construction, and then the database construction was carried out according to the sample size for database construction.
Data collection	Zeyu Li collected ATAC-seq data, Mengdi Li collected RNA-seq data and four epigenetic sequencing data (DNA methylation, H3K4me3, H3K27me3, and H3K27ac). We used a various of software to analyze the raw data, and record the process and results of data analysis.
Timing and spatial scale	The temporal and spatial scale of data collection was not involved in this study. We controlled the collected samples to be in the same growth state, and then conducted database sequencing, and data analysis and data collection on the sequencing results. This process did not involve the timing and spatial scale of data collection.
Data exclusions	No data were excluded from analyses.
Reproducibility	All attempts to repeat the experimental were successful.
Randomization	Uniformly growing plant material was randomly selected for leaves.
Blinding	Data collection in this study was obtained through software analysis of data, and there was no subjective bias in this process, so blinding was not relevant to our study.

Did the study involve field work? ☐ Yes ☒ No

Reporting for specific materials, systems and methods

We require information from authors about some types of materials, experimental systems and methods used in many studies. Here, indicate whether each material, system or method listed is relevant to your study. If you are not sure if a list item applies to your research, read the appropriate section before selecting a response.

Materials & experimental systems

n/a	Involved in the study
☐	☒ Antibodies
☒	☐ Eukaryotic cell lines
☒	☐ Palaeontology and archaeology
☒	☐ Animals and other organisms
☒	☐ Clinical data
☒	☐ Dual use research of concern

Methods

n/a	Involved in the study
☐	☒ ChIP-seq
☒	☐ Flow cytometry
☒	☐ MRI-based neuroimaging

Antibodies

Antibodies used	H3K4me3(cat. no. ab8580,abcam); H3K27me3(cat. no. 9733,CST); H3K27ac(cat. no. ab4729,abcam).
Validation	The primary antibodies used were chosen based on already published data with the relevant citations in the manuscript.

ChIP-seq

Data deposition

☒ Confirm that both raw and final processed data have been deposited in a public database such as GEO.

☒ Confirm that you have deposited or provided access to graph files (e.g. BED files) for the called peaks.

Data access links<br>*May remain private before publication.*	https://www.ncbi.nlm.nih.gov/Traces/study/?acc=SRP299560&o=acc_s%3Aa
Files in database submission	Input and IP of H3K4me3, H3K27ac, H3K27me3 of natural B. napus L. (cv. Darmor), resynthesized B. napus L. (HC-2), B. rapa L. (cv. 9JC002), and B. oleracea L. (cv. 3YS013).
Genome browser session<br>(e.g. UCSC)	https://www.genoscope.cns.fr/brassicanapus/cgi-bin/gbrowse/colza/

Methodology

Replicates	Young leaves of five-week-old plants were harvested with three biological replicates.
Sequencing depth	All experiments were sequenced at a depth of 10G and the sequencing strategy was PE150 (paired-end 150 bp). The total number of reads and uniquely mapped reads of A_H3K27ac_IP were 59,756,256 and 41,777,111. The total number of reads and uniquely mapped reads of A_H3K27ac_Input were 70,310,980 and 37,534,130. The total number of reads and uniquely mapped reads of A_H3K27me3_IP were 65,818,134 and 38,071,100. The total number of reads and uniquely mapped reads of A_H3K27me3_Input were 70,379,386 and 37,546,026. The total number of reads and uniquely mapped reads of A_H3K4me3_IP were 63,137,314 and 46,606,226. The total number of reads and uniquely mapped reads of A_H3K4me3_Input were 66,994,758 and 35,700,042. The total number of reads and uniquely mapped reads of C_H3K27ac_IP were 64,602,192 and 48,572,663. The total number of reads and uniquely mapped reads of C_H3K27ac_Input were 83,680,010 and 55,395,647. The total number of reads and uniquely mapped reads of C_H3K27me3_IP were 63,592,548 and 41,385,094. The total number of reads and uniquely mapped reads of C_H3K27me3_Input were 63,863,280 and 42,183,149. The total number of reads and uniquely mapped reads of C_H3K4me3_IP were 66,959,250 and 52,765,788. The total number of reads and uniquely mapped reads of C_H3K4me3_Input were 64,856,566 and 42,616,666. The total number of reads and uniquely mapped reads of NAC_H3K27ac_IP were 71,569,610 and 59,267,149. The total number of reads and uniquely mapped reads of NAC_H3K27ac_Input were 75,939,058 and 50,555,216. The total number of reads and uniquely mapped reads of NAC_H3K27me3_IP were 65,569,976 and 44,512,729. The total number of reads and uniquely mapped reads of NAC_H3K27me3_Input were 65,664,658 and 43,449,513. The total number of reads and uniquely mapped reads of NAC_H3K4me3_IP were 62,434,844 and 52,956,174. The total number of reads and uniquely mapped reads of NAC_H3K4me3_Input were 64,372,808 and 42,582,295. The total number of reads and uniquely mapped reads of RAC_H3K27ac_IP were 64,976,692 and 49,520,052. The total number of reads and uniquely mapped reads of RAC_H3K27ac_Input were 66,459,800 and 40,888,157. The total number of reads and uniquely mapped reads of RAC_H3K27me3_IP were 60,846,134 and 40,205,588. The total number of reads and uniquely mapped reads of RAC_H3K27me3_Input were 63,910,424 and 39,271,256. The total number of reads and uniquely mapped reads of RAC_H3K4me3_IP were 65,794,168 and 51,272,420. The total number of reads and uniquely mapped reads of RAC_H3K4me3_Input were 69,853,812 and 43,017,251.
Antibodies	H3K4me3(cat. no. ab8580,abcam); H3K27me3(cat. no. 9733,CST); H3K27ac(cat. no. ab4729,abcam).
Peak calling parameters	epic --treatment {ip_beds} --control {input_beds} --number-cores {threads} --outfile {output} --bed {bbeedd} --chromsizes {genome_size} --false-discovery-rate-cutoff 0.00001 --effective-genome-fraction 1 --log {log_file}
Data quality	Q30 of clean data were more 98% and the ratio of clean read and raw read was approximately 90%. A total of 62,603 peaks were at FDR 5% and above 5-fold enrichment.
Software	FastQC, Trimmomatic, STAR, MACS2, bedtools, edgeR.
